# *Drosophila* Tribbles Antagonizes Insulin Signaling-Mediated Growth and Metabolism via Interactions with Akt Kinase

**DOI:** 10.1371/journal.pone.0109530

**Published:** 2014-10-15

**Authors:** Rahul Das, Zachary Sebo, Laramie Pence, Leonard L. Dobens

**Affiliations:** Division of Molecular Biology and Biochemistry, School of Biological Sciences, University of Missouri-Kansas City, Kansas City, Missouri, United States of America; Institute of Molecular and Cell Biology, Singapore

## Abstract

*Drosophila* Tribbles (Trbl) is the founding member of the Trib family of kinase-like docking proteins that modulate cell signaling during proliferation, migration and growth. In a wing misexpression screen for Trbl interacting proteins, we identified the Ser/Thr protein kinase Akt1. Given the central role of Akt1 in insulin signaling, we tested the function of Trbl in larval fat body, a tissue where rapid increases in size are exquisitely sensitive to insulin/insulin-like growth factor levels. Consistent with a role in antagonizing insulin-mediated growth, trbl RNAi knockdown in the fat body increased cell size, advanced the timing of pupation and increased levels of circulating triglyceride. Complementarily, overexpression of Trbl reduced fat body cell size, decreased overall larval size, delayed maturation and lowered levels of triglycerides, while circulating glucose levels increased. The conserved Trbl kinase domain is required for function in vivo and for interaction with Akt in a yeast two-hybrid assay. Consistent with direct regulation of Akt, overexpression of Trbl in the fat body decreased levels of activated Akt (pSer505-Akt) while misexpression of trbl RNAi increased phospho-Akt levels, and neither treatment affected total Akt levels. Trbl misexpression effectively suppressed Akt-mediated wing and muscle cell size increases and reduced phosphorylation of the Akt target FoxO (pSer256-FoxO). Taken together, these data show that *Drosophila* Trbl has a conserved role to bind Akt and block Akt-mediated insulin signaling, and implicate Trib proteins as novel sites of signaling pathway integration that link nutrient availability with cell growth and proliferation.

## Introduction

Tribbles (Trib) family members are found throughout the metazoan lineage and in mammals have multiple roles in development, tissue homeostasis and disease, where they have been identified as oncogenes and tumor suppressors, depending on the tissue context [1], [2]. Trib function was first characterized in the *Drosophila* embryo, where Tribbles (Trbl) binds and degrades String/Twine phosphatase to block cell division both early during the midblastula transition and later in the invaginating mesoderm during gastrulation [3]–[6]. In the fly ovary, Trbl binds and degrades the C/EBP homolog Slow Border Cells (Slbo) to modulate cell migration [7]. Trib family members share three conserved motifs: (1) a divergent kinase-like domain notably lacking key residues required for catalytic activity, (2) a ubiquitin ligase COP1 binding domain and (3) a site for binding the MAP kinase kinase MEK1 [8], [9]. A growing list of Trib targets, including diverse kinases and transcription factors, has led to the proposal that these are non-functional pseudokinases that act as adaptor molecules to bind and block the activity of key regulatory molecules, effectively balancing levels of multiple signaling pathways to coordinate cell differentiation, proliferation and growth [10].

To identify novel components in the Tribbles signaling pathway, we have undertaken a misexpression screen in the fly wing, and here we report an interaction between fly Tribbles and Akt1, a key regulator of the insulin signaling pathway. The insulin/insulin-like growth factor (IGF) signaling (IIS) pathway is conserved throughout the metazoan lineage and functions to sense local and systemic nutrient levels and connect this information to the control of cellular and organismal metabolism [11]. Insulin signaling regulates tissue homeostasis, longevity and diverse developmental processes including body size and sexual maturation [12]. Insulin and insulin-like peptides act in an endocrine manner to bind insulin receptors (InR) in responsive tissue [13]–[15]. This triggers a phosphorylation cascade from the insulin receptor substrate (IRS) to phosphoinositide-3 kinase (PI3K [16]), which promotes the conversion of phosphatidylinositol 4,5-bisphosphate (PIP2) to phosphatidylinositol 3,4,5-triphosphate (PIP3) in the cell membrane [17]. PIP3 recruits phosphoinositide-dependent protein kinase 1 (PDK1 [18]) and Akt/PKB kinase [19], [20]. Akt is activated by phosphorylation at Thr308 and Ser473 (equivalent of *Drosophila* Ser505) and Akt in turn phosphorylates myriad substrates to promote cellular anabolism, including: (1) the Rheb-specific GTPase activating protein (GAP) Tsc2 to promote TOR Complex 1 (TORC1) signaling and protein synthesis [21]–[23]; (2) GSK-3β to block glucose production and stabilize MYC to boost anabolic gene expression [24], [25]; and (3) the transcription factor FoxO to block its nuclear localization and reduce expression of catabolic genes [26].

The strength and duration of insulin signaling is controlled by phosphatases [27] and feedback phosphorylation [28]–[30]. Recently, mammalian Trib3 and Trib2 have been demonstrated to bind Akt and block its activation without lowering Akt levels, resulting in impaired insulin signaling in hepatocytes, adipocytes, skeletal muscle, liver, fat, and pancreas [31]–[36]. Consistent with a role in reducing insulin outputs, increased Trib3 expression occurs (1) following either starvation or exercise in mice, (2) in *db/db* diabetic mice [37] and (3) following experimental treatments such as high-fructose feeding or chronic ethanol consumption that lead to impaired insulin responses [38], [39]. Aberrantly high Trib3 levels are detected in insulin-resistant humans [33], [40], [41] and a population variant Trib3Q/R84 associated with predisposition to metabolic disease dominantly blocks insulin signaling in cell culture [42]–[44].

The notion that Trib3 binds Akt to ‘dial-down’ the insulin response in peripheral tissues is contradicted by genetic analysis showing that a rat Trib3 knockdown has no effect on Akt activity [45] and mouse Trib3 knockout has no effect on metabolism at all [46], phenotypes that may be due to overlapping functions with Trib1 and Trib2. Evidence shows that Tribs influence metabolism via effects on lipogenesis, as well: (1) Trib3 binds and degrades Acc1 to reverse fatty acid deposition [32]; (2) Trib1 modulates hepatic lipogenesis [47]; (3) sequence variations in humans Trib1 are associated with increased plasma lipoproteins and the risk of coronary artery disease [48]–[50]; and (4) Tribs block adipocyte differentiation and triglyceride accumulation by inhibiting the transcriptional activity of three master regulators of fat cell differentiation – PPARγ (inhibited by Trib1), MLXIPL (inhibited by Trib1) and C/EBPα (inhibited by Tribs 1, 2 and 3 [51]–[54]).

*Drosophila* presents a model system to test how insulin and insulin-like growth factor signaling (IIS) regulate tissue growth, glucose homeostasis, lipid metabolism fecundity and longevity [55]. Genes and tissue interactions comprising the fly insulin pathway have strong sequence, structural and functional homology to members of the vertebrate pathway [56]. Mutations in fly IIS pathway components result in reduced cell, organ and body size with little effect on cell fate and differentiation. Similarly, ablation of the fly insulin-producing cells in the brain, analogous to the pancreatic beta-cells, leads to decreased animal size and increased sugar concentrations, modeling dwarfism and type 1 diabetes, respectively [57]. Also, many of the insulin target tissues in *Drosophila* are functionally equivalent with adipose, liver, brain, kidney and skeletal muscle, all known to be important in type 2 diabetes [58], [59]. The parallels between flies and vertebrates extends to diet so that flies raised on high sugar diets feature the hallmarks of insulin resistance, including hyperglycemia and increased fat [60]–[62].

Here we show that *Drosophila* Trbl negatively regulates insulin signaling during tissue growth by binding and blocking the phosphorylation-dependent activation of Akt, underscoring the putative function of Trib proteins as central nodes in multiple signaling pathways regulating cell growth, proliferation and differentiation, and raising the intriguing possibility that Tribs may have a conserved role in coordinating nutrient-dependent growth in development and disease.

## Results

### Akt interacts with Trbl in a wing misexpression screen

**Figure 1:**
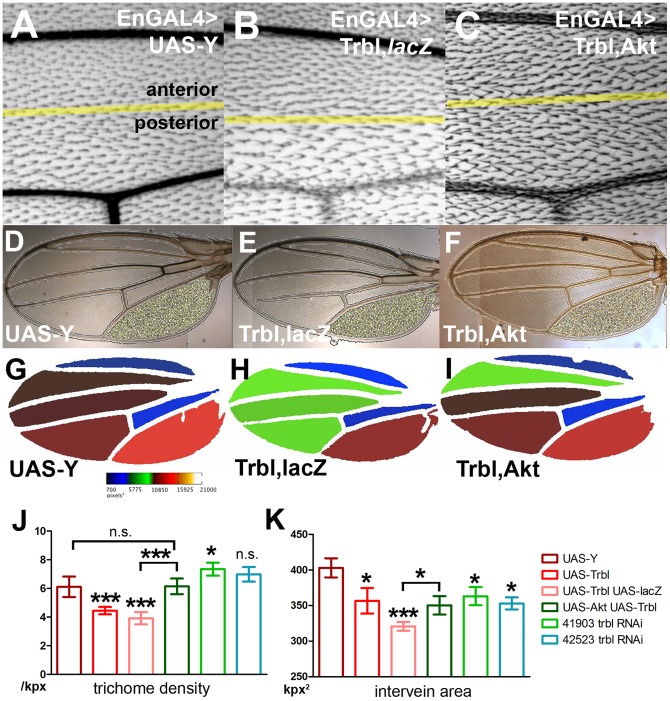
Akt is a Trbl interacting gene in a wing misexpression screen. (A) engrailedGAL4>UAS-Y control wing shows normal size and trichome distribution in both the anterior and posterior compartments (genotype enGAL4>UAS-Y). (B) engrailedGAL4>UAS-Trbl misexpression in the posterior compartment shows a reduced trichome density relative to WT (genotype enGAL4>UAS-*lacZ*, UAS-Trbl). (C) The Trbl misexpression trichome phenotype is suppressed by coexpression of Akt, restoring the normal distribution of trichomes (genotype enGAL4>UAS-Akt, UAS-Trbl). (D-K). Fijiwings analysis of Trbl and Akt misexpression in wing. (D–F) Detection of trichomes for representative wings. (G–I) Heat map analysis of intervein areas for representative wings. Note that third posterior intervein region in WT is red, brown in Trbl misexpressing wing and the same area is reddish brown following co-expression of Akt. (J) Measurement of trichome density (in trichomes per kilopixel) is the result of the average of at least four wings for each genotype. Trbl misexpression in posterior wing compartment resulted in an 18.3% decrease in trichome density, an effect that was not lessened by co-misexpression of a UAS-*lacZ* transgene, indicating UAS-transgene dosage did not modify Trbl phenotypes, and this reduction was antagonized by co-misexpression of UAS-Akt. For J and K, for average trichome densities, n = 8 for UAS-Y and UAS-Trbl, 10 for UAS-Trbl UAS-*lacZ* and UAS-Akt; UAS-Trbl, 6 and 4 for 41903 and 42523 RNAis respectively. P values calculated from one way ANOVA (for the entire group) and from two-tailed paired t test (between two genotypes where indicated) are summarized in Table S1 (n.s. =  not significant; *P<0.05; **P<0.01; ***P<0.001), and all error bars are ± SD. (K) Measurement of area (kpx, in kilopixels of third posterior intervein region) is the average of at least three wings for each genotype. Trbl misexpression in posterior wing compartment resulted in a 12% decrease in intervein tissue size, an effect that was not suppressed by co-misexpression of a UAS-*lacZ* transgene, indicating UAS-transgene dosage did not modify Trbl phenotypes and this reduction was effectively antagonized by co-misexpression of UAS-Akt. For areas, n = 3 for all cases except for 42523 RNAi where n = 2.

Trbl misexpression blocks cell proliferation in the embryo and wing, consistent with its ability to bind String/Twine cdc42 phosphatase and block entry into mitosis [4], [5]. As a consequence, misexpression of Trbl in the posterior wing compartment using the engrailedGAL4 driver results in an increase in cell size (detectable as a decrease in the density of trichomes, which mark individual wing cells; Fig. 1B,E,J) and a decrease in overall wing size (Fig. 1E,H,K [63]). We performed a wing co-misexpression screen for genes that modify these divergent Trbl misexpression phenotypes (Anna Shipman, R.D. and L.L.D., in preparation) and identified Akt1, which suppressed Trbl phenotypes when co-misexpressed with Trbl, effectively decreasing cell size and increasing tissue size to generate a wing structure that appeared nearly wild type (Fig. 1C,F,I-K).

To precisely document the interaction between Trbl and Akt, we used Fijiwings, an automated program, to both measure posterior intervein area and calculate trichome density, in this regions [63]. Because trichomes are produced by individual wing epithelial cells, trichome density is an inverse measure of cell size. In WT wings, trichome density was 6.1 trichomes/square kilopixels (kpx^2^; averaged from at least three WT wings) while Trbl misexpression reduced trichome density in the posterior intervein region to 4.5 trichomes/kpx^2^. The strength of this phenotype was not diluted by co-misexpression of UAS-*lacZ* (3.5/kpx^2^; Fig. 1J), whereas co-misexpression of Trbl with Akt increased the density to 6.1, a significant increase resulting in a trichome density similar to WT (Fig. 1J). With respect to tissue size, WT wings had an average area of 402 kpx^2^ in the posterior intervein region, and Trbl,*lacZ* misexpression reduced this to 320.8 kpx^2^ while co-misexpression of Trbl with Akt resulted in an area of 350.2 kpx^2^, a significant increase in size (Fig. 1K).

### Trbl antagonizes cell growth in larval tissue

**Figure 2:**
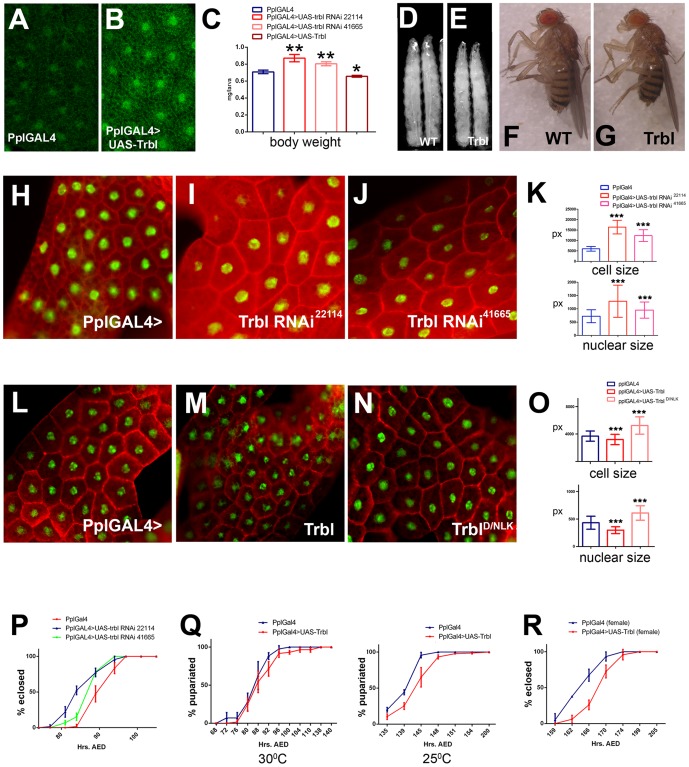
Larval fat body specific overexpression of Trbl suppresses growth and delays development. (A,B). Trbl antisera detects a cytosolic and nuclear antigen in mid-3^rd^ instar larval fat body (A) whose levels increase in PplGAL4>UAS Trbl larvae (B; note that images in A and B were taken under identical fixation and microscopy conditions). (C). Body weight measurement of age matched mid-3rd instar larvae of indicated genotypes. Compared to the control, misexpression of trbl RNAi line 22114 increased body weight by ≈25% and trbl RNAi line 41665 increased body weight by ≈14% (in triplicate, n = 16/genotype) whereas Trbl overexpression in fat body reduced larval weight by ≈7%. (D,E). Age matched mid 3^rd^ instar control PplGAL4 larvae (D, designated WT) are larger than PplGAL4>UAS Trbl larvae (E). (F,G). Adult body size comparison of two day-old females is shown. PplGAL4>UAS Trbl female adults (F) are smaller than PplGAL4 control (G, designated WT). (H–J). Size analysis of micrographs of fat body cells from age-matched mid 3^rd^ instar larvae is shown. PplGAL4 control (H) and two RNAi lines (I,J) are shown. Tissues were stained with phalloidin (red) to reveal actin at cell periphery and DAPI (green) to reveal nuclear size. Note that Fig 2H–J and 2L–N are taken under the same magnification, collectively. (K). Measurement of cell size (top) and nuclear size (bottom) of fat body cells expressing Trbl RNAi. Quantification of size is made in pixels. For K, n = ∼50 for cell size and n = >90 for nuclear size. P values from T test are indicated (n.s. =  not significant; *P<0.05; **P<0.01; ***P<0.001) and are summarized in Table S1 and all error bars are ±S.D. (L–N). Size analysis of micrographs of fat body cells from age-matched mid 3^rd^ instar larvae is shown. PplGAL4 control (L), Trbl (M) and Trbl^D/NLK^ (N) are shown. Tissues were stained with phalloidin (red) to reveal actin at cell periphery and DAPI (green) to reveal nuclear size. Note that these experiments were performed at 25°C because PplGAL4>UAS-D/NLK is lethal at 30°C. (O). Measurement of cell size (top) and nuclear size (bottom) of fat body cells expressing Trbl or Trbl^D/NLK^. n = ∼100 for cell size and ∼100 nuclear size except for UAS-Trbl where n = 52. (P). Misexpression of both Trbl RNAi lines 22114 and 41665 advanced pupariation compared to a PplGAL4 age matched control. For P-R, all error bars are ±S.D. (Q). Pupariation, measured by eversion of spiracles in wandering larva, is delayed in Trbl overexpressing larvae compared to age-matched control PplGAL4 larvae reared at 30°C (panel 1) and this difference is accentuated when development is slowed at 25°C (panel 2; each performed in triplicate, n≈25/genotype). (R). Eclosion of PplGAL4>UAS Trbl females is delayed relative to an age matched control (performed in triplicate, n≈10/genotype).

Akt1 is a key mediator of insulin signaling, so we focused on the requirement for Trbl in the larval fat body, an insulin responsive tissue [64]. Specific antisera revealed fat body expression of Trbl localized to the nucleus and more diffuse in the cytoplasm (Fig. 2A), and we confirmed the specificity of the Trbl antisera by overexpressing UAS-Trbl using the fat body-specific Pumpless-GAL4 (Ppl-GAL4) driver, which resulted in a strong increase in detectable nuclear and cytoplasmic Trbl staining compared to controls (Fig. 2A,B [65]).

To reduce *trbl* levels in the fat body, we used several RNAi lines (see Materials and Methods) that were effective in suppressing Trbl wing phenotypes when co-expressed in wing tissue using the en-GAL4 driver (data not shown). While these lines had no strong effect on wing growth when misexpressed alone (Fig. 1J,K and data not shown), fat body misexpression of UAS-trbl RNAi resulted in a significant increase in both larval weight (Fig. 2C) and fat body cell size (Fig. 2H–K) relative to control tissue. Because larval size and weight thresholds trigger pupariation and eclosion, we measured the timing of these events and observed an advance in the timing of pupariation in trbl RNAi-overexpressing larva compared to WT controls (Fig. 2P, panel 1), consistent with this increase in weight.

Compared to WT larva, Ppl-GAL4 misexpression of Trbl in the fat body reduced significantly body weight in age-matched larvae (Fig. 2C), and reduced overall size of both age-matched larvae (cf. Fig. 2D,E) and two-day old adult females (Fig. 2F,G), though males were not appreciably affected (data not shown). Direct examination of the larval fat body tissue confirmed that UAS-Trbl overexpression reduced cell and nuclear size significantly compared to WT (Fig. 2L,M,O). The ability of Trbl to reduce fat body cell size was dependent on an intact kinase domain, as misexpression of a Trbl transgene bearing a site directed mutation in the conserved ATP binding motif in the divergent kinase domain (D/NLK [66]) increased cell size significantly compared to WT (Fig. 2N), as measured by cell circumference or nuclear size (Fig. 2O). These comparisons (Fig. 2L–O) were made at 25°C because Trbl^D/NLK^ caused lethality when misexpressed at 30°C, perhaps due to dominant effects of high levels of this mutant version (data not shown; see discussion).

Overexpression of Trbl in the larval fat body delayed the timing of pupariation compared to a wild type control cohort (Fig. 2Q, panel 1). This delay was more marked at 25°C where growth periods are longer (Fig. 2Q, panel 2). These changes in the timing of pupariation following Trbl overexpression suggest larvae feed an extended period to reach critical weight before pupa formation, and consistent with this, overexpression of Trbl at 30°C led to a delay in eclosion, to a greater extent in females than males (Fig. 2R).

**Figure 3:**
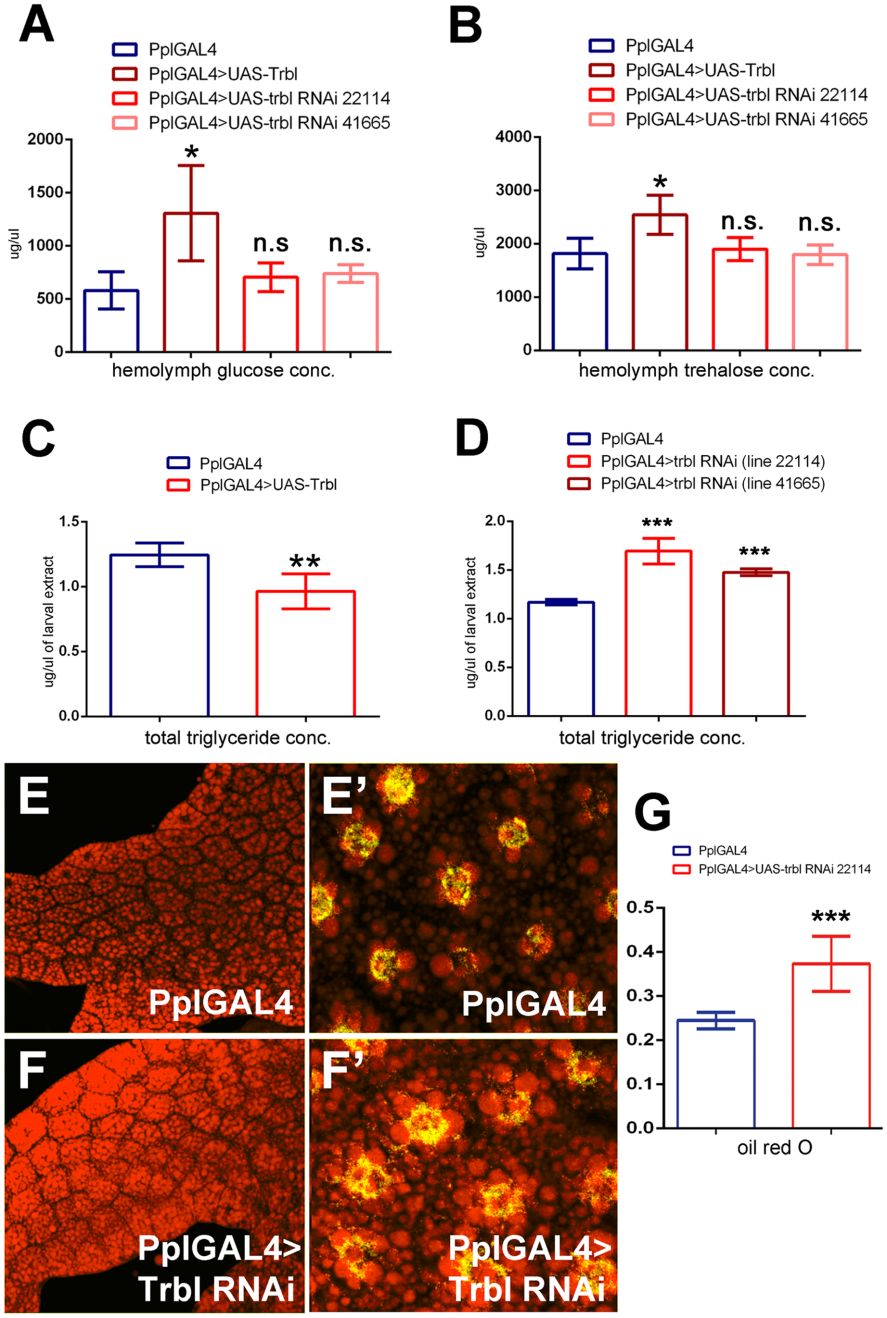
Trbl affects circulatory sugar and total lipid level. (A). Compared to controls, Trbl overexpression in fat body increases hemolymph glucose level by ≈110% whereas trbl RNAi knock down does not alter glucose level significantly (in triplicate, n = 30/genotype). For panels A,D and G, n = 3 and n = 6 for C and P values from T test are indicated (n.s. =  not significant; *P<0.05; **P<0.01; ***P<0.001) and are summarized in Table S1 and all error bars are ± S.D. (B). Compared to controls, hemolymph trehalose levels were significantly increased in larvae overexpressing Trbl in fat body but did not change significantly in trbl RNAi knock down larvae. (in duplicate, n = 30/genotype). (C). Compared to controls, overexpression of Trbl reduces total triglyceride level significantly by ≈22%. n = 6. (D). Compared to controls, misexpression of trbl RNAi line 22114 increased significantly total triglyceride level by ≈44% and for trbl RNAi line 41665 triglyceride levels were increased significantly by ≈25%. n = 3. (E,F). Trbl RNAi misexpression increases lipid accumulation. Nile red staining of dissected WT tissue (E,E′, DAPI in green) shows reduced lipid accumulation compared to fat body misexpressing Trbl RNAi (F,F′, line 21114). n = 3. (G). Fat body from PplGAL4 flies driving expression of Trbl RNAi (line 22114) shows a significant increase in Oil Red O binding (in arbitrary units) compared to fat body tissue dissected from control PplGAL4 flies. n = 3.

We next examined the effect of manipulating Trbl levels on metabolites (Fig. 3). Compared to WT larva, Trbl overexpression in the fat body led to a significant increase in circulating levels of both glucose (Fig. 3A) and trehalose (Fig. 3B), the main circulatory sugar in *Drosophila*, a glucose disaccharide synthesized from intracellular glucose and secreted from the fat body. In contrast, trbl RNAi misexpression had little effect on levels of either glucose or trehalose compared to control animals (Fig. 3A,B). Compared to WT larva, Trbl overexpression led to a significant decrease in total triglyceride levels (Fig. 3C), the main form of stored fat in wandering larva, while trbl RNAi misexpression in the fat body resulted in a significant increase in triglyceride levels (Fig. 3D [60]). Consistent with increased triglycerides, trbl RNAi misexpression in the fat body resulted in a noticeable increase in the number and size of lipid drops as revealed by fluorescence from Nile Red staining compared to WT tissue (cf. Fig. 3E,F). To measure more precisely this increase in lipid, we isolated fat body tissue expressing trbl RNAi and compared the extent of Oil RedO binding to control tissue. As shown in Fig. 3G, trblRNAi^22114^ line showed a significant increase in absorbance at 510 nm compared to controls corresponding to the increased dye bound to lipid in this tissue (Fig. 3G). These data are consistent with previous reports showing that reduced insulin signaling leads to hypoglycemia and decreased accumulation of fat [60] and point to a conserved role for Trbl in blocking insulin signaling to reduce growth and increase catabolic pathway activity.

### Trbl suppresses Akt-mediated larval growth phenotypes

**Figure 4:**
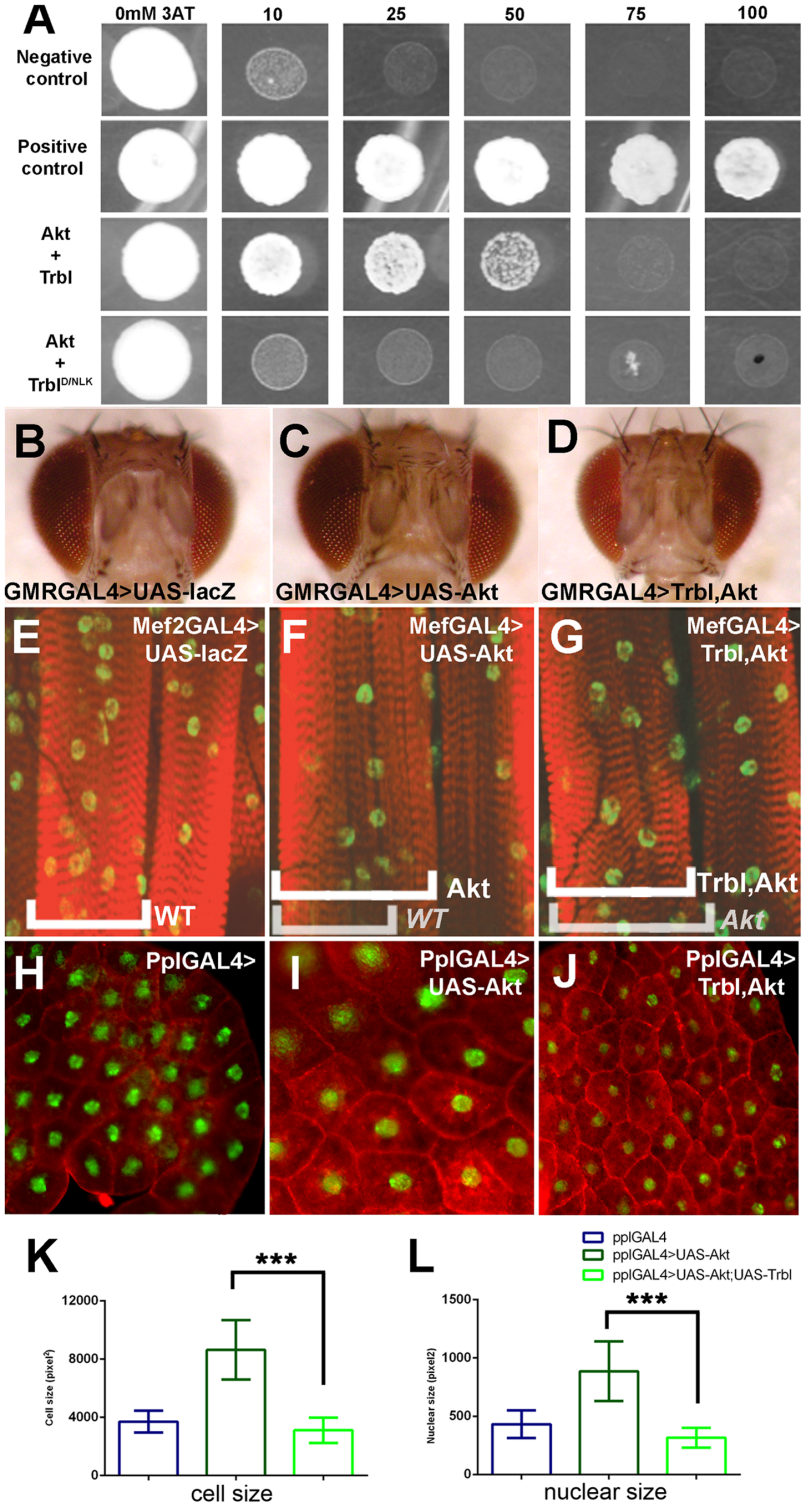
Trbl binds Akt and suppresses Akt-mediated cuticle growth phenotypes. (A). The ability of yeast cells to grow on increasing concentrations of 3AT growth inhibitor depends on the strength of the protein-protein interaction. Yeast cells co-expressing Akt prey and WT Trbl bait are able to grow in presence of up to 50 mM 3AT whereas yeast cells co-expressing Akt and Trbl D/NLK bait are unable to grow in presence of 10 mM 3AT, similar to negative controls. (B–D). Akt misexpression in head capsule by the GMRGAL4 driver results in increased head size (C), which is effectively suppressed by Trbl co-misexpression (D). Genotypes: (B) GMRGAL4, (C) GMRGAL4>UAS-Akt1, (D) GMRGAL4>UAS-Akt1, UAS-Trbl. (E–G). Akt misexpression in the larval muscle using the MefGAL4 driver results in an increase in muscle size (cf. E,F) while Trbl effectively suppresses this (G). Genotypes: (E) Mef2GAL4, (F) Mef2GAL4>UAS-Akt1, (G) Mef2GAL4>UAS-Akt1, UAS-Trbl. (H–J). Age matched mid 3rd instar larval fat body cells from PplGAL4>UAS-Akt (I) are detectably larger that WT (H). This Akt-mediated increase in cell size is noticeably suppressed in fat body cells co-expressing Trbl and Akt (J). Tissues were stained with Phalloidin (red) to reveal cell boundary and DAPI (green) to reveal nucleus. Note that Fig 4B-D, E-G, H-J are taken under the same magnification, respectively. (K). Quantification of fat body cell size in pixels (n≈100 cells/genotype; for K and L, P values from two-tailed T test data are indicated (n.s. =  not significant; *P<0.05; **P<0.01; ***P<0.001) and are summarized in Table S1 and all error bars are ± S.D. (L). Quantification of fat body nuclear size in pixels (n≈100 nuclei/genotype).

To understand the molecular nature of Trbl's interaction with Akt, we performed a yeast two-hybrid assay, expressing Trbl in the bait vector and Akt1 in the prey vector. As shown in Fig. 4A, Trbl interacts with Akt1, resulting in detectable growth under stringent conditions (up to 50 mM 3-Amino-1,2,4-triazole on HIS^-^ dropout plates). A mutation in the conserved kinase domain (Trbl^D/NLK^) reduced growth to levels comparable to a negative control, consistent with a disruption in this interaction with Akt (Fig. 4A).

We examined next Trbl interactions with Akt during growth and patterning of larval tissues (Fig. 4B–L). Akt1 misexpression in the eye and head using the GMR-GAL4 driver increased head size compared to WT (Fig. 4B,C). While GMR-GAL4 misexpression of Trbl results in no change from the WT head size (data not shown), co-misexpression of Trbl and Akt effectively suppressed this Akt large head phenotype (cf. Fig. 4C,D). In the larval muscle (Fig. 4E), Mef2-GAL4 misexpression of Akt at 25°C led to an increase in the breadth of muscle fibers (Fig. 4F) that was reduced by co-misexpression of Trbl (Fig. 4G). Interestingly, at 30°C, Mef2-GAL4 driving Akt was lethal, a phenotype that was suppressed by Trbl co-misexpression (data not shown). Finally in the fat body (Fig. 4H), Ppl-GAL4 misexpression of Akt1 resulted in a large cell phenotype (Fig. 4I), which was suppressed by co-misexpression of Trbl (Fig. 4J). Akt-mediated increases in fat body cell and nuclear size were significantly reduced by Trbl co-misexpression (Fig. 4K and L, respectively). Thus, Trbl can effectively inhibit Akt-mediated growth in different types of larval tissue, i.e. mitotic and endoreplicating tissues.

**Figure 5:**
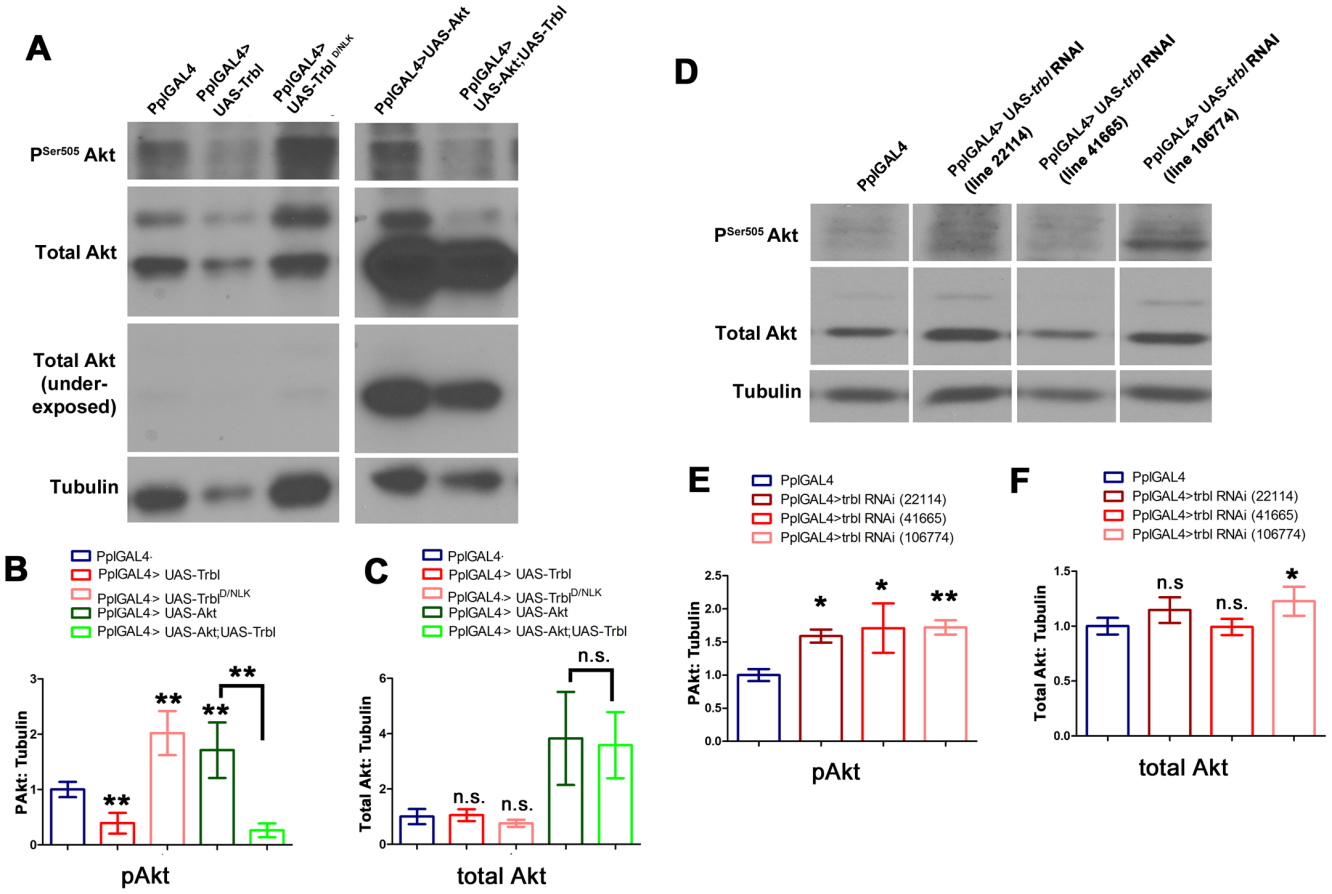
The Trbl kinase domain is required to block Akt activation. (A). Representative Western blot of fat body from age matched 3^rd^ instar larvae driving transgene expression by PplGAL4. In panels A and D, fat body extract from identical numbers of larva was loaded in each lane. See text for details. (B,C) Quantification of Western blots of fat body extracts from four independent (n = 4) experiments showing the effect of Trbl and Trbl^D/NLK^ on Akt activation and total Akt levels. For quantification of panels B,C,E and F, α-tubulin band was used as loading control and results were normalized to PplGAL4; P values from on-way ANOVA and two-tailed T test data are indicated (n.s. =  not significant; *P<0.05; **P<0.01; ***P<0.001) and are summarized in Table S1 and all error bars are ± S.D. (B). pSer^505^ Akt. (C). Total Akt. (D). Representative Western blot of fat body extract from age matched 3^rd^ instar larvae driving transgene expression by PplGAL4. (E,F). Quantification of western blots of fat body extracts from four independent experiments showing the effect of trbl RNAi knock-down on Akt activation. (E). pSer^505^ Akt. (F). Total Akt.

Next, we examined the effect of Trbl overexpression on the activation of Akt and total Akt level using antisera specific to each. We probe Western blots of protein extracts collected from dissected fat bodies, and measured protein levels from scanned blots of at least four independent experiments (Material and Methods, Fig. 5 and Supplemental Data Figure 1). Representative blots presented in Fig. 5A and summarized in Fig. 5B,C show that Ppl-GAL4 driving Trbl led to a significant reduction of phospho-Akt levels compared to control animals; in contrast, levels of total Akt were not significantly different from control animals when corrected for levels of tubulin, which were consistently reduced in Trbl-misexpressing larva, likely die to the overall reduction in animal size (Fig. 5A,C). In contrast, misexpression of the kinase dead mutation Trbl^D/NLK^ resulted in an increase in phospho-Akt levels compared to control animals without affecting total Akt levels (Fig. 5A–C), consistent with the increase in cell size following Trbl^D/NLK^ misexpression (Fig. 2N,O).

As expected, ectopic Akt led to a strong increase in both phospho-Akt and total Akt, confirming the specificity of these antisera (Fig. 5A–C). Trbl co-misexpression with Akt was sufficient to inhibit significantly phosphorylation-dependent activation of Akt, resulting in a 6.5-fold decrease compared to Akt misexpression (Fig. 5A,B). As expected, Trbl had no effect on the high levels of total Akt produced by Akt co-misexpression (Fig. 5A,C). We note that Trbl co-misexpression did not affect the levels of total Akt compared to levels seen following misexpression Akt alone (Fig. 5C), confirming that UAS-transgene dosage does not titrate Ppl-GAL4. As shown in Fig. 5D-F, misexpression of three independent trbl RNAi lines led to a significant increase in endogenous phospho-Akt levels with no significant change in total Akt levels (in two of three RNAi lines used; Fig. 5D–F).

### Trbl blocks insulin signaling upstream but not downstream of Akt

**Figure 6:**
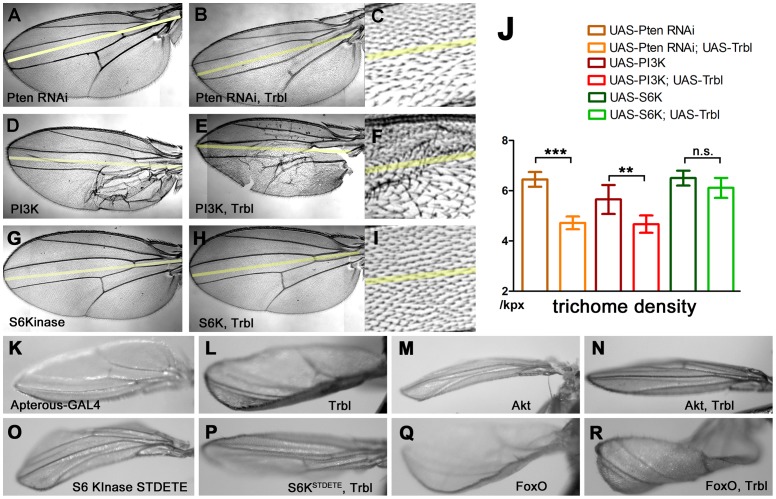
Trbl blocks insulin signaling. (A) engrailedGAL4>UAS-PtenRNAi misexpression. (B,C) enGAL4>UAS-PtenRNAi, UAS-Trbl co-misexpression results in the Trbl large cell phenotype. (D) engrailedGAL4>UAS-PI3K misexpression results in posterior wing patterning defects. (E,F) enGAL4>UAS-PI3K,UAS-Trbl co-misexpression results in the Trbl large cell phenotype. (G) engrailedGAL4>UAS-S6Kinase. (H,I) enGAL4>UAS-S6Kinase,UAS-Trbl co-misexpression effectively suppresses the Trbl large cell phenotype. (J) Trichome densities from genetic interactions shown in A-I. Average trichome densities from n = 6 wings; P values from one-way ANOVA and T tests between two genotypes where indicated are summarized in Table S1 (n.s. =  not significant; *P<0.05; **P<0.01; ***P<0.001) and all error bars are ± SD. (K) apterousGAL4 control results in flat wing blade. (L) apterousGAL4>UAS-Trbl causes upturned wings, (M) apterousGAL4>UAS-Akt results in downturned wings, (N) apterousGAL4>UAS-Akt, UAS-Trbl results in a flat wing blade. (O) apterousGAL4>UAS-S6KTEDE results in downturned wings. (P) apterousGAL4>UAS-S6KTEDE, UAS-Trbl suppresses the activated S6kinase effect on the wing, resulting in a flat wing blade. (Q) apterousGAL4> UAS-FoxO results in upturned wing. (R) apterousGAL4>UAS-FoxO, UAS-Trbl results in strongly upturned wings.

Our data are consistent with the notion that Trbl can block insulin signaling by binding and preventing Akt phosphorylation-dependent activation, without affecting Akt levels. Because the insulin pathway is highly conserved in *Drosophila*, we tested the epistatic relationships between Trbl and components of insulin signaling lying upstream and downstream of Akt using the misexpression of transgenes in the wing (Fig. 6). As shown previously in Fig. 1, engrailed-GAL4 misexpression of Trbl in the posterior compartment of the wing resulted in a decrease in trichome density, a phenotype upon which we focused on for our analysis here. En-GAL4 misexpression of an RNAi targeting Pten, an upstream inhibitor of insulin signaling [67], increased trichome density as shown previously [63], and this Pten RNAi phenotype was suppressed by Trbl co-expression, resulting in a significant decrease in trichome density (Fig. 6B,C and J). Similarly, Trbl misexpression reduction in trichome density was not masked by PI3 kinase, when co-misexpressed in the posterior wing (Fig. 6E,F and J). In contrast, En-GAL4 misexpression of a wild type copy of S6 kinase, which lies downstream of Akt, effectively suppressed the Trbl large cell phenotype, resulting in an average trichome density not significantly different from S6 kinase expression alone (Fig. 6H,I and J).

It has been shown previously that apterous-GAL4 misexpression of insulin signaling components results in dorsal compartment overgrowth leading to a characteristic bending of the wing [23], and the tissue specificity of this driver allowed us to test Trbl interaction with several Akt targets that were lethal in combination with en-GAL4. apterous-GAL4 misexpression of Trbl caused the wing to bend up, consistent with the notion that Trbl blocks cell division and growth when misexpressed in the dorsal wing compartment (Fig. 6J,K [4]). In contrast, ap-GAL4 misexpression of Akt caused the wing to bend downward (Fig. 6M). This Akt overgrowth phenotype was suppressed by co-misexpression of Trbl, resulting in a nearly flat wing blade (Fig. 6N).

**Figure 7:**
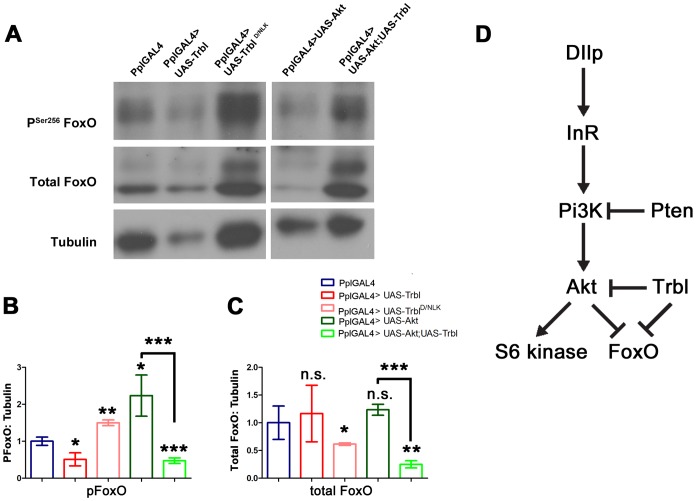
Trbl reduces FoxO phosphorylation; a model for Trbl action. (A). Representative Western blot of fat body extract from age matched 3^rd^ instar larvae driving transgene expression by PplGAL4. Equal amount of fat body extract was loaded in each lane. Tubulin blot picture is same as Fig. 5A because the same blot was stripped and reprobed as described in materials and methods. See text for details. (B,C). Quantification of western blots of fat body extracts from four independent experiments (n = 4) showing the effect of Trbl and Trbl^D/NLK^ on FoxO phosphorylation and total FoxO levels. See text for details. For all quantification, α-tubulin was used as loading control and results were normalized to PplGAL4; P values from one-way ANOVA and two-tailed T test data are indicated (n.s. =  not significant; *P<0.05; **P<0.01; ***P<0.001) and are summarized in Table S1 and all error bars are ± S.D. (B). PSer^256^ FoxO. (C). Total FoxO. (D). Model for Trbl regulation of Akt and FoxO. See text for details.

We next tested interactions between Trbl and two Akt targets. ap-GAL4 misexpression of an activated version of the *Drosophila* homolog of the Akt target S6 kinase 1 (*dS6KSTDE* [68]) resulted in a bending downward of the wing blade (Fig. 6N), an overgrowth phenotype that was effectively suppressed by Trbl co-expression (Fig. 6O), consistent with combinatorial, opposing interaction between these genes. Misexpression of FoxO, a target of Akt phosphorylation-dependent inhibition, led to a curved upward wing (Fig. 6P), a phenotype that was noticeably enhanced by Trbl co-misexpression (Fig. 6Q), suggesting an additive interaction between Trbl and FoxO to block growth. To examine Trbl effects on FoxO directly, we used specific antisera to detect Akt phosphorylation-inactivation of FoxO, which occurs at Ser 256. We probed Westerns of fat body extracts from age matched Trbl-overexpressing larvae using antisera to phospho-FoxO and used a pan-FoxO antisera to simultaneously detect effects on total FoxO levels. As before, we performed at least four experiments and scanned protein levels from multiple autoradiographic exposures. As shown in representative Westerns presented in Fig. 7A-C, Trbl overexpression in the fat body led to a significant reduction in phospho-FoxO levels compared to controls (Fig. 7A,B), while total FoxO levels were unaffected when corrected for total protein levels as measured by tubulin, which, as noted before, was consistently reduced in Trbl-misexpressing animals (Fig. 7A,C). Akt misexpression led to a significant increase in phospho-FoxO, indicating that the antisera can reliably detect Akt-dependent phosphorylation-dependent inactivation of FoxO (Fig. 7A,B). Trbl co-misexpression effectively blocked this Akt-dependent increase in phospho-FoxO levels. Unexpectedly co-misexpression of Trbl and Akt in the fat body led to a significant reduction in total FoxO levels (Fig. 7A,C), suggesting that Trbl and Akt might act combinatorially to direct FoxO turnover. Finally, we detected a reproducible and significant increase in phospho-FoxO levels following misexpression of Trbl^D/NLK^ (Fig. 7A,B), an increase that parallels the increase in phospho-Akt levels associated with misexpression of Trbl^D/NLK^ (Fig. 5E–G). Despite this increase in phospho-FoxO levels, Trbl^D/NLK^ misexpression led to a significant decrease in total FoxO levels (Fig. 7A,C), an unexpected effect that, again, may be due to dominant effects on endogenous Trbl as discussed below.

## Discussion

Previously, Trbl has been shown to regulate activities of the C/EBP Slbo during cell migration and in separate work, Trbl has been shown to regulate activity of String/Twine during cell proliferation [4], [5], [7], in both cases by binding the respective proteins and directing their proteasome-dependent degradation. Here we show that *Drosophila* Trbl binds Akt to block its phosphorylation-dependent activation and does so without affecting Akt levels (model in Fig. 7D). By this mechanism, fly Trbl blunts insulin signaling responses to regulate anabolic and catabolic pathways affecting circulating and stored metabolites with influences on body size, weight and the timing of both pupariation and eclosion. Here we discuss some key details of our work and more generally, the role of Tribbles and Trib family members as conserved sites of integration for pathways regulating tissue morphogenesis, cell division and metabolism.

Our demonstration that a mutation in the kinase-like domain of Trbl (D/NLK) reduces the ability of Trbl to bind Akt and block Akt-mediated cell growth is consistent with previous work showing that this mutation disrupts the ability of Trbl to block Slbo-dependent cell migration and direct Slbo/C/EBP turnover [66]. Despite the requirement for the kinase-like domain in mediating Trbl function, several of our observations indicate that Trbl^D/NLK^ retains effects on metabolic activity: (1) misexpression of Trbl^D/NLK^ led to an increase in fat body cell size; (2) Trbl^D/NLK^ misexpression increased Akt and FoxO phosphorylation; and (3) surprisingly, Trbl^D/NLK^ was lethal when strongly misexpressed in the fat body. While it is possible that Trbl^D/NLK^ interferes with endogenous Trbl to promote Akt activity, our yeast two hybrid (Y2H) data shows that both Trbl-Trbl and Trbl-Akt interactions are disrupted by the D/NLK mutation [66]. Thus, the dominant effects of Trbl^D/NLK^ occur in a manner that is indirect or requires endogenous factors absent in the Y2H assay, possibilities that we are testing now.

Our misexpression studies show that WT Trbl effectively blocks insulin signaling both upstream and at the level of Akt. Downstream from Akt, S6 kinase co-misexpression suppressed the Trbl curved-wing phenotype and FoxO co-misexpression enhanced it. When we examined FoxO protein, we found Trbl reduced FoxO phosphorylation without an effect on total FoxO levels, an outcome that is expected if Trbl blocks Akt activation. Co-misexpression of Trbl and Akt reduced FoxO phosphorylation as well, but unexpectedly reduced significantly total FoxO levels relative to misexpression of either transgene alone (Fig. 7C). This outcome is reproducible and suggests that Akt and Trbl act synergistically to direct FoxO degradation (model, Fig. 7D). Another puzzling result came from examining the effect of Trbl^D/NLK^ misexpression on FoxO: while an increase in FoxO phosphorylation occurred, which is expected given that Trbl^D/NLK^ increases Akt activity, Trbl^D/NLK^ potently reduced total FoxO levels. This decrease in total FoxO levels makes the simultaneous significant increase in phospho-FoxO following Trbl^D/NLK^ misexpression even more striking. In humans, Trib2 has been identified as a repressor of FoxO in malignant melanomas [69] and complimentarily, FoxO represses Trib3 expression to derepress Akt and enhance insulin sensitivity in hepatocytes [70]. Thus there is good reason to expect significant cross-regulation among insulin, Akt, FoxO, and Tribbles, which we are currently testing using tools provided in the fly model.

Our demonstration that Trbl binds and blocks Akt activation without affecting Akt levels indicates functional conservation with mammalian Trib3, which was identified in a yeast two hybrid screen of Akt1 interactors and was shown to block phosphorylation-dependent activation of Akt, in a manner that required the conserved kinase domain [31], [39]. Though some evidence contradicted these early findings [71], the notion that Trib3 regulates insulin signaling was bolstered by the identification of a human Q84R SNP in Trib3 associated with insulin resistance and type 2 diabetes. When this population variant was introduced into a Trib3 transgene and misexpressed in cell culture, Q84R was shown to reduce Akt phosphorylation more potently, possibly by enhanced binding to AKT2 [42], [43].

The Trib family of proteins mediates a highly complex network of molecular and cellular interactions, yet how Tribs link these diverse pathways to allow proper cell function is poorly understood. In mice, Trib3 regulates lipogenesis by triggering the degradation of acetyl-CoA carboxylase via association with an E3 ubiquitin ligase [32] and mediates the cellular endoplasmic reticulum stress response [72]–[75], suggesting this family of proteins integrates metabolic and inflammatory pathways in various tissues. In mice and humans, Trib3 levels are regulated by nutrient availability in skeletal muscle cells, beta-cells, adipocytes, and tumor cells, highlighting a possible role in the regulation of metabolic flux in insulin-sensitive tissues [33], [76]–[79].

Since Tribs likely act as adaptor proteins to regulate multiple cell signaling pathways and key cell cycle components, it is unsurprising that they been associated with colorectal cancers [80], lung cancer [81], breast cancer [82], melanoma [69], acute myeloid leukemia [83], [84], esophageal carcinogenesis [85] and chronic lymphocyte leukemia [86]. Despite a growing body of work featuring Trib proteins' roles in cancer and metabolic disorders, a mechanistic understanding of the pathway disruptions underlying disease physiology is far from complete. That our data strongly support a role for Trbl in blocking Akt activity should dispel confusion generated by contradictory results obtained from mammalian systems, and substantiate *Drosophila* as a well-equipped genetic model organism to dissect Trib function in the regulation of metabolism and growth.

## Materials and Methods

### Drosophila strains

(1) P{UAS-FLAG-trbl.WT} (UAS-FlagTrbl) and (2) P{UAS-FLAG-trbl.DNLK} (UAS-FlagTrbl^D/NLK^) have been described previously [66]. (3) P{w[+mC] = GAL4-Mef2.R}3 (Mef2-GAL4) was a gift from Erika Geisbrecht and (4) P{Ppl-GAL4.P} was a generous gift from John B. Thomas of Salk Institute. We obtained the following stocks from the Indiana Stock Center, (5) P{en2.4- GAL4}e16E (engrailed-GAL4, en-GAL4), (6) P{GawB}ap[md544] (apterous-GAL4, ap-GAL4), (7) P{longGMR-GAL4}3 (8) y[1] w[1118]; P{w[+mC] = UAS-Akt1.Exel}2, (9) P{UAS-Pi3K92E.Exel}2, (10) P{UAS-S6k.STDE}3, (11) P{UAS-foxo.P}2; (12) P{TRiP.JF01859}attP2; from the Vienna stock center, (13) w[1118]; P{GD11640}v22114; w[1118]; (14) P{KK108667} (106774); and from the Harvard stock center (15) P{TRiP.HMS02198} (41665).

### Yeast two-hybrid screen

Construction of bait vectors FLAGTRBL and FLAGTRBL^D/NLK^ and their use in yeast two-hybrid analysis was described previously [66]. To construct prey plasmid Akt1 cDNA was amplified using PCR to add flanking attB1 and attB2 sites. Forward and reverse primers were: **GGGGACAAGTTTGTACAAAAAAGCAGGCTTCA** TGTCAATAAACACAACTTTCGACCTCAGCTC and GGGG**ACCACTTTGTACAAGAAAGCTGGGTC**CTATTGCATCGATGCGAGACTTGTG (attB1 and attB2 sequences in bold). The PCR product was cloned into the donor vector pDONR-21 and then into either pDEST32 (bait vector) or pDEST22 (prey vector). All constructs were confirmed by DNA sequencing.

### Quantification of metabolites and lipids

Flies were reared on “Cornmeal, Molasses and Yeast Medium” (Bloomington Stock Center recipe). Measurements of the glucose and trehalose concentration in the hemolymph and triglyceride content of larval extracts were performed as described in [60]. For total triglyceride quantification, ∼10 age matched larvae/group were washed three times in PBS, then wiped briefly on Kimwipe. Larvae were transferred to a 1.5 ml Eppendorf tube and homogenized on ice in 40 ul of ice cold PBST (0.1% Tween20)/larva with a hand held homogenizer on ice. Homogenates were then heated at 65°C for ∼5 minutes to deactivate lipases. 20 ul of the homogenate was added to tube containing 680 uL of triglyceride reagent (Thermo Infinity Triglycerides Reagent), incubated at 37°C for 15 minutes. Samples were then centrifuged briefly to remove suspended particles and then measured on a plate reader at 540 nm in biological triplicate. For Nile Red stains, larvae were dissected on a glass slide in 0.001% Nile Red (Sigma), 75% glycerol and incubated for 10 minutes before mounting. Slides were imaged as described previously [60]. For Oil red O assay, dissected fat body was incubated with a working solution of 0.5% Oil Red O dye in 6 ml isopropanol+ 4 ml H2O (prepared freshly and filtered through a 0.45 µm filter), incubated for 30 minutes at room temperature, washed 3 times in Milli Q H_2_O and then the tissue bound dye was extracted with 100% isopropanol. O.D. was measured at 510 nm.

### Western blots and immunostaining

Age matched larval fat body was homogenized in 2x sample buffer and equal amounts of homogenate were loaded onto SDS–PAGE gels and blotted according to standard protocol. Blots were probed with the following antibodies (all at 1∶2000 dilution): (1) phospho-*Drosophila* Akt (Ser505) Antibody (Cell Signaling #4054), (2) Akt (pan) (C67E7) Rabbit mAb (Cell Signaling #4691), (3) anti Phospho-FoxO1 Ser256 (Cell Signaling #9461), (4) anti-dFOXO (a generous gift from Marc Tartar), (5) 12G10 anti-alpha-tubulin (Developmental Studies Hybridoma Bank). Four experiments were done and multiple exposures taken; Western blot quantification was done on lightly exposed blots using Licor Image analysis software. Blots were stripped and reprobed using mild stripping protocol of Abcam (http://www.abcam.com/ps/pdf/protocols/stripping%20for%20reprobing.pdf). Primary antibodies were used in the following order - P-Akt, P-FoxO, total Akt, Total FoxO, α-tubulin. Secondary antibodies conjugated to HRP (Genescript) were used, and the signals were detected by chemiluminescence using the Enhanced ECL kit (Biorad).

For whole-mount immunostaining of larval body wall muscles, age matched samples were collected and washed 3 times with PBS in an Eppendorf tube. Then they were incubated with 65°C PBS for 30 seconds. After that, the hot PBS was replaced with ice cold PBS and the tube was placed on ice. This technique straightens the larvae and makes dissected muscle flat. The larvae were laid in ice cold PBS on their dorsal side and dissected along the anterior-posterior axis. Fat body and other tissues were removed before the samples were fixed for 20 min in PBS with 4% paraformaldehyde. After washing three times in PBS+0.1%Tween 20 (PBST), samples were incubated for 30 min with Phalloidin (1∶2000, Sigma), washed 3 times in PBST (15 minutes each time) and then mounted on 50% glycerol with DAPI (1∶1000, Roche). Image analysis was done with ImageJ and Photoshop. Micrographs were collected using an Olympus confocal laser-scanning microscope and figures prepared using Photoshop. Wing preps were made as previously described [63]. Wing curvature was documented on thoracic shish kabobs photographed using a Nikon dissection scope and attached Nikon D100 camera. Cell size and nuclear size was documented using the tools in Fijiwings [63].

### Body size analysis

For wing analysis, crosses were reared at 30°C and female wings were selected and mounted. For Fijiwings analysis, we used FijiwingsEZ and calculated wing size in kilo pixels and trichome density in “per kilo pixel.” For analysis of body weight, age matched groups of mid L3 larvae were weighed on a precision Kahn microbalance (model 4400). More than 10 age matched larvae/group were washed three times in PBS, dried on Kimwipe and weighed in triplicate and the average body weight calculated. Wing areas and trichome densities were measured as previously described [63].

### Larval fat body analysis

Approximately 10 age matched mid third instar female larvae were collected in ice cold PBS and dissected partially in PBS to open up the body cavity and then fixed in 4% paraformaldehyde in PBS for 20 minutes. Fixed tissue was washed for 3 times in PBST (PBS+0.1% Triton X100, 10 minutes each time) and incubated with blocking reagent (PBST+10% normal goat serum) for 2 H at room temperature with rocking. Then the samples were washed 3 times in PBST (5 minutes) and incubated with 1° antibody (1∶1000 chicken anti-Trbl in PBST+2.5% BSA) overnight at 4°C with rocking. Then the tissue was washed 3 times in PBST (10 minutes) and incubated with 2° antibody (goat anti chicken Alexa Fluor488 conjugated IgG) in PBST+2.5% BSA) for 2 hours at room temperature with rocking. Tissue was washed 3 times in PBST (10 minutes) and once with PBS. Then a single strip of fat body from the ventral mid-section of each larva was collected and mounted on 75% glycerol to visualize the fluorescent staining.

For actin and DAPI staining, tissue was fixed and blocked as described above and washed for 3 times in PBST (5 minutes) and incubated with 1∶500 TRITC conjugated Phalloidin (from Roche) in PBST+2.5% BSA for 20 minutes at room temperature with rocking. Then the samples were washed 3 times in PBST (10 minutes) and once with PBS. Then a single strip of fat body from the ventral mid-section of each larva was collected and mounted on 75% glycerol+20 µM DAPI (Roche, 10236276001) to visualize fluorescent staining. A single image was taken from each fat body strip (hence each larva) and multiple images were used for the quantification.

### Larval age synchronization and pupation time assay

∼40 virgin (2–5 days old) and male flies were reared on “Cornmeal, Molasses and Yeast Medium” (Bloomington Stock Center recipe) supplemented with dry yeast for three days. Food was changed every day. On day four, flies were transferred to a fresh vial for 30 min to get rid of any held eggs. After that, flies were again flipped to a new vial with regular food (no yeast supplement) and kept for 2 hours. Females laid ∼20 eggs during that time. After 18 hrs, all unhatched eggs were removed from the vial. Larvae were collected from that vial at proper developmental time for assays. Mid 3^rd^ instar larvae were selected from food and presence of food in the gut was checked. For pupation assay, ca. 30 first instar larvae were plated in triplicate on regular fly food at 30°C or 25°C. Pupae formed were counted in every 4–8 hours over the course of 6–7 days. After 7 days, total number of pupae was counted and assigned as 100%. The fraction of the larvae pupariated during different time was calculated from total pupariated. Experiment was done in biological triplicate.

For the eclosion assay, ∼30 synchronized wandering 3^rd^ instar larvae were collected and transferred to the wall of a new vial. The number of pupae hatched was counted in every few hours. After 7 days, total number of pupae counted and assigned as 100%. The fraction of the male/female animals hatched during different time was calculated from total animals hatched. Experiment was done in biological triplicate.

### Statistical Analysis

Statistical analysis was performed with Graph Pad Prism (v 5.0) and all data are presented as mean ± SD and analyzed using one way ANOVA and/or unpaired, 2-tailed Student's t test (as detailed in Table S1). A P-value of less than 0.05 is considered significant.

## Supporting Information

Table S1 **P value calculations and results for** **Figs 1** **-** **7** **.**
(XLSX)
