# Correction: *Drosophila* Tribbles Antagonizes Insulin Signaling-Mediated Growth and Metabolism via Interactions with Akt Kinase

**DOI:** 10.1371/journal.pone.0123150

**Published:** 2015-04-02

**Authors:** The PLOS ONE Staff

There is an error in the labeling of Fig. 7A. Please see the corrected Fig. 7 here.

**Fig 7:**
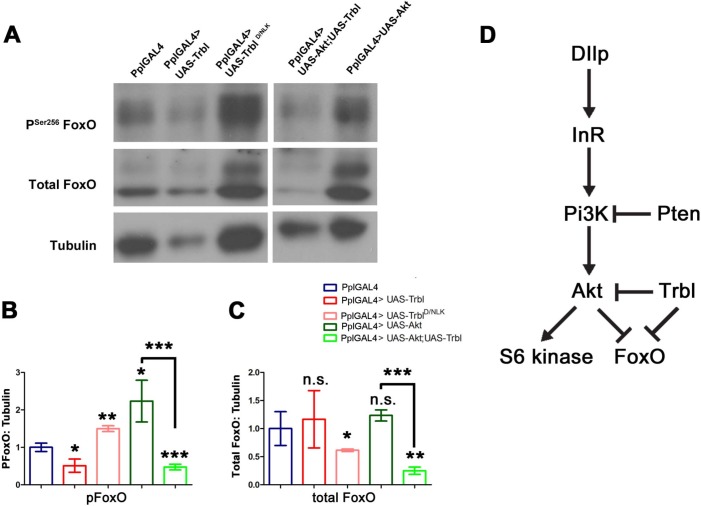
Trbl reduces FoxO phosphorylation; a model for Trbl action. (A). Representative Western blot of fat body extract from age matched 3^rd^ instar larvae driving transgene expression by PplGAL4. Equal amount of fat body extract was loaded in each lane. Tubulin blot picture is same as Fig. 5A because the same blot was stripped and reprobed as described in materials and methods. See text for details. (B,C). Quantification of western blots of fat body extracts from four independent experiments (n = 4) showing the effect of Trbl and Trbl^D/NLK^ on FoxO phosphorylation and total FoxO levels. See text for details. For all quantification, α-tubulin was used as loading control and results were normalized to PplGAL4; P values from one-way ANOVA and two-tailed T test data are indicated (n.s. = not significant; *P<0.05; **P<0.01; ***P<0.001) and are summarized in Table S1 and all error bars are ± S.D. (B). PSer^256^ FoxO. (C). Total FoxO. (D). Model for Trbl regulation of Akt and FoxO. See text for details.
